# Structure and species composition of diatom community during the wet season in three floodplain lakes of Brazilian Pantanal

**DOI:** 10.1371/journal.pone.0251063

**Published:** 2021-05-05

**Authors:** Margaret S. Nardelli, Denise C. Bicudo, Silvio C. Sampaio, Cláudia M. d. S. Cordovil

**Affiliations:** 1 Programa Engenharia Agrícola, Recursos Hídricos, Universidade Estadual do Oeste do Paraná, Cascavel, Paraná, Brasil; 2 Núcleo de Ecologia, Instituto de Botânica, SIMA, São Paulo, Brasil; 3 Centro de Estudos Florestais, CEF, Instituto Superior de Agronomia de Lisboa, Universidade de Lisboa, Lisboa, Portugal; University of Nottingham, UNITED KINGDOM

## Abstract

In order to access environmental conditions, the use of bioindicators that have a close relationship with environmental stressors is a largely common practice, but when evaluating environmental inferences, the individual dominant taxa need to be interpreted. Humid regions such as the marshlands are fragile ecosystems and sustain communities of microalgae, often used as bioindicators, of which diatoms are a good example. Although they provide an excellent response to chemical and physical changes in water, diatom studies in surface sediments in wetlands are scarce worldwide. To determine whether diatom species have the potential to provide unambiguous inferences in the influence of environmental factors, we have evaluated diatom abundance in surface sediment, from three Pantanal lakes, against a set of environmental gradients: pH, dissolved oxygen, turbidity, conductivity, total dissolved solids, water temperature, index of trophic water status, total phosphorus and total nitrogen. The Ferradura lake presented an oligotrophic state and both Burro and Caracará lakes presented mesotrophic state. Diatoms were more abundant in the a mesotrophic conditions, but with higher species richness in the oligotrophic conditions. Depending on the N:P ratio, the nutrients nitrogen and phosphorus can also play the role of pollutants and may have negative and unpredictable effects in the environment, such as biotic homogenization. Despite the spatial variation in species, there was a greater richness of *Eunotia* Ehrenberg species, with the highest relative density of *Eunotia formica* Ehrenberg and *E*. *pantropica* Glushchenko, Kulikovskiy & Kociolek, due to the environmental acidic conditions, a determining characteristic of this genus. It was also observed that a small increase in the level of phosphorus generated an increase in the abundance of *Aulacoseira* Thwaites with the highest relative density of *A*. *pusilla* (Meister) Tuji & Houki and *A*. *veraluciae* Tremarin, Torgan & T.Ludwig. However, *A*. *italica* dominated in the moderately acidic environment. The results can help with decisions in impacted areas to solve socioeconomic problems, environmental management and biodiversity.

## Introduction

Aquatic environments have frequently been affected by different anthropic activities, resulting in negative impacts to river basins in developed [1–6] and wild regions [7–9], such as Pantanal of Mato Grosso, the largest continuous floodplain in South America, located in Brazil. Compared to the six Brazilian continental biomes, the Pantanal Biome accounts for only 1.76% of the country territory. However, it is of outstanding importance due to the complexity of habitats and high diversity of plant and animal species, and therefore is considered a World Natural Heritage and Biosphere Reserves by Unesco [10].

Moreover, it is well known for its annual flood pulse, a river-plain interaction which affects the entire biota of the system [11]. For example, the natural eutrophication phenomenon locally called *Decoada*, which occurs during the beginning of the flood phase, causes a series of changes in water quality that are of great importance to the processes of decomposition and chemosynthesis [12].

Another important impact is the anthropogenic eutrophication, aggravated in wetlands by the annual floods [13,14], which harms the structure and dynamics of the communities of aquatic organisms [12]. The increase in nutrient concentration during the eutrophication process, drastically changes the microorganism biomass populations [15]. Robust conclusion of environmental condition may be drawn from the presence of bioindicators that have intense relationships with stressors, such as the diatoms [16]. Nevertheless, the interpretation of individual dominant taxa, needs to be addressed when making environmental inferences [17–19].

Diatoms are a group of silicified microalgae, considered as one of the most sensitive groups to environmental changes [20]. In the last decades, the study of diatom assemblages, linked to any single substratum, has received increasing attention [21–26], because it provides relevant information about the ecosystem stratification, allowing a correlation of ecological information with time and space [27–29], as well as the assessment of the ecological status of rivers, streams and lakes in temperate zones [30–32]. However, there is still an urgent need to expand the information to the wetland regions of the globe, where studies of diatom community are scarce [33,34]. So far, in South America, namely in Brazil, studies have focused on the planktonic and epilithic diatoms in rivers and streams, mainly related to the evaluation of water quality [35–37] and periphytic diatoms in floodplain [38]. Recent studies have focused on the role of eutrophication in environmental reorganization, with diatom assemblages and land-use records used as a tool to infer the trophic state history of the water body [39,40] as well as a record of biotic homogenization of diatom diversity in sedimentary samples [41]. Furthermore, some advances were also made in the auto-ecology of tropical species, the influence of environmental and spatial factors on diatom biodiversity, and its distribution [42,43].

In spite of these advances, there are a lack of studies on diatoms in surface sediment. Taxonomic studies contribute to the knowledge of biodiversity and provide the basis for the advancement of other approaches such as bioindication, environmental reconstruction, research on conservation and definition of priority areas such as the Pantanal, among many others. The more relevant works in the region are on the distribution of two species of diatoms and their association with the historical variation in water levels in the Paraná River [44]; the diatom flora in the Pantanal of Mato Grosso do Sul [45]; the history of the salinity in the southern Pantanal [46] and records of flood pulse dynamics [47]. Other studies, not related to diatoms, refer to the wetland carbon storage [48] and the influence that hydroclimatic variables exert on limnogeological processes [49]. However, the diatom biodiversity of this region remains understudied. This may be due to the difficulty of sampling in flooded areas as a result of a lack of suitable transportation through the wetland, as well as people specialized in diving in these areas. Thus, to better understand the biodiversity of diatoms in sediments of tropical wetland areas, the present study aimed to evaluate the influence of environmental factors on the distribution of these organisms in surface sediments in three different lakes of the Brazilian Pantanal (wetland) of Mato Grosso State. Our approach merges an assessment of diatom species with presence/absence and relative abundance data.

This study brings a contribution to the understanding of flooded tropical regions and intends to increase the knowledge of diatom biodiversity in Pantanal, using the structure and composition of species as a limnological bioindicator in tropical wetlands that are still poorly explored. Furthermore, the results of this study in the current context of the environmental destruction of Pantanal (fire/forest 2020) and the influence that it may have had on limnological processes, currently and also in the future, are extremely relevant to raise new comparative studies for the area, to help with decisions that may be of socioeconomic reasons, environmental management and also of biodiversity issues.

## Materials and methods

### Ethics statement

The Surface sediment and water samples were taken from public water bodies, no location was on protected or private land. No permits were required for the described study. The field sampling was done in accordance with Brazil national and regional regulations and permission was not necessary to collect the data. The field studies involved neither endangered nor protected species.

### Study area

In this study, the surface sediments of three permanent lakes of the Pantanal of Mato Grosso were collected in 10 sites per lake (Table 1) and analysed for the diatom species present, as a bioindicator of the state of water quality.

**Table 1:** Geographic coordinates of the sampling sites in the studied lakes.

Ferradura lake			Burro lake			Caracará lake		
Sites/zone	Lat S	Lon W	Sites/zone	Lat S	Lon W	Sites/zone	Lat S	Lon W
1-inflow	16°31’35"	56°23’26"	11-inflow	17°50’24"	57°23’53"	21-inflow	17°53’32"	57°27’55"
2-marginal	16°31’33"	56°23’25"	12-marginal	17°50’22"	57°23’44"	22-marginal	17°53’42"	57°27’19"
3-middle	16°31’32"	56°23’26"	13-marginal	17°49’17"	57°24’04"	23-middle	17°53’11"	57°27’24"
4-marginal	16°31’25"	56°23’30"	14-middle	17°49’00"	57°23’49"	24-middle	17°52’31"	57°27’29"
5-middle	16°31’23"	56°23’31"	15-middle	17°48’46"	57°23’18"	25-marginal	17°52’11"	57°27’44"
6-middle	16°31’22"	56°23’35"	16-middle	17°47’30"	57°23’27"	26-middle	17°51’31"	57°27’16"
7-marginal	16°31’21"	56°23’40"	17-middle	17°46’40"	57°22’54"	27-middle	17°51’07"	57°27’40"
8-middle	16°31’18"	56°23’47"	18-middle	17°46’15"	57°22’39"	28-middle	17°50’50"	57°27’46"
9-outflow	16°31’23"	56°23’56"	19-outflow	17°46’08"	57°22’37"	29-outflow	17°50’33"	57°27’44"
10-outflow	16°31’24"	56°23’54"	20-outflow	17°45’46"	57°22’27"	30-outflow	17°50’29"	57°27’53"

The Pantanal is located on a 140,000 km^2^ plain in the tropical southwest of Brazil, bordering Bolivia and Paraguay, with coordinates 15° to 22° S and 55° to 58° W [50]. The flooded area, to the North and Northeast, is the source of the large rivers Paraguai, São Lourenço and Cuiabá, which are responsible for the flooding of the Pantanal North and for conditioning the floods along the North-South axis of the Paraguai river [12]. The predominant conditions in Pantanal determine a period of intense summer rain (November to March) with periods of high water and flooding, and other season the of dry (April to October) [51]. The average annual atmosphere temperature is around 25° C, ranging from a maximum of 34° C to a minimum of 15° C [52]. Average annual rainfall is 1400 mm, with a variation between 800 and 1600 mm, with 70% during the rainy season, November to March [53].

### Characterization of the experimental sites

The three lakes studied were:

- Ferradura Lake (FP), located at 16°31’24” S and 56°23’40” W, with an average width of 300 m, an approximate extension of 1200 m, depth of 270–650 cm and connected to the Cuiabá river. Rainfall data (accumulated monthly) from five months prior to collection in the Ferradura lake region, weather station code: 1656002, see Table 2 [54].
- Burro Lake (BP), located at 17°45’46” S and 57°23’44” O, with an average width of 1000 m, an approximate extension of 5000 m, depth of 140–280 cm and connected to the São Lourenço river. Rainfall data (accumulated monthly) from five months prior to collection in the Burro lake region, weather station code: 1655001, see Table 2 [54].
- Caracará Lake (CP) located at 17°50’33” S and 57°27’52” O, has an average width of 3000 m with approximate extension of 3600 m, depth of 120–290 cm and connected to the Paraguai river. Rainfall data (accumulated monthly) from five months prior to collection in the Caracará lake region, weather station code: 1757001, see Table 2 [54].

**Table 2:** Rainfall data from five months prior to collection for region the of three areas studying.

Rainfall data	Station code	Oct/2014	Nov/2014	Dec/2014	Jan/2015	Feb/2015
Ferradura lake	1656002	26 mm	237 mm	125 mm	144 mm	321 mm
Burro lake	1655001	58 mm	136 mm	322 mm	106 mm	195 mm
Caracará lake	1757001	33 mm	99 mm	159 mm	62 mm	170 mm

In the literature it is found that the Cuiabá river is influenced by the discharge of sewage as well as by pisciculture that release their effluents containing high total N levels. Both São Lourenço and Paraguai rivers are influenced by agricultural, livestock activities as well as by heavy metals mainly by mercury, due to gold mining activities [12].

### Sampling method and analysis of samples

Collection of superficial sediment (SS) was performed in February 2015. This month is considered to be representative due to high water from the flooding characteristic of summer rains. For the analysis of diatoms taxa ten samples of SS were collected with an Ekman dredger in the first 5.0 cm at the bottom of each lake, in the inflow, at the outflow, in the middle and in the marginal zone. Water samples were collected in polyethylene bottles on the sub-surface water of the lakes from the same 10 points for physical and chemical analysis. Both sample sets were kept under refrigeration for three days (2±0.5 °C).

Each sediment sample (0.5 g) was oxidized according to the standard method [55] using 35% H_2_O_2_ and 37% HCl. After the cleaning process, the slides (1 ml of the oxidized) were prepared with NAPHRAX, for qualitative and quantitative analysis of the different diatom taxa found. For the registration of the species (qualitative analysis) an image capture microscope (Zeiss Axioskop 2 plus), equipped with a digital camera (DC500) of high resolution, with a magnification of up to 1000 × was used.

For the identification of the species, at the lowest possible taxonomic level, classical ecological floras and new published data were used [56–63]. Counting of slides of 400 diatom valves (quantitative analysis) was performed to verify the relative density [28]. Diatom analysis was performed on species that achieved relative abundances ≥5% on at least one sampling station. Diatom species codes were assigned according to the Omnidia software [64].

Water temperature, pH, dissolved oxygen, turbidity (TU), conductivity, total dissolved solids (TDS), and depth were obtained with multiparameter probe (Horiba U50). Water total P and total N were determined according to Valderrama [65] and chlorophyll-*a* analysis followed Marker [66] method.

The water trophic state index was established according to Lamparelli [67], adopting values of trophic classification for lentic environment for chlorophyll-*a* and total P. Analysis of the TSI (Trophic State Index) is a measure of the potential of eutrophication, with P being the nutrient considered to be the causative agent.

N:P “Redfield ratio” was also determined. It is an important indicator in water bodies, indicating which nutrient is probably limiting productivity. Thus, the nutrient that will limit the growth of phytoplankton is the nutrient that reaches a minimum value before the other nutrients [68].

### Statistical analysis

Differences between means were verified by ANOVA and the f-test, for both abiotic (physical-chemical) and biotic parameters (species frequency). Pearson’s linear analysis was also performed with species that presented abundance greater than 5%, as well as Pearson analysis to verify the correlation between its abiotic variables. Principal Component Analysis (PCA) was performed with the abiotic and also with biotic variables, the PCA allows you to identify patterns in the data and express them in such a way that their similarities and differences are highlighted.

In order to deal with multiple factors, biological (diatom distribution), chemical and physical variables, a multivariate statistical analysis was performed using canonical correspondence analysis (CCA). For the CCA analysis, only the biotic variables with abundances ≥5% and that showed autocorrelation (p <0.05, Pearson) were selected. For the abiotic variables, the conductivity and total dissolved solids variables were extracted, which are variables related to turbidity. The analysis was performed using the program XLstat 2018.1.01.

## Results

### Abiotic variables

Chemical and physical characterization of the lakes is presented in Table 3.

**Table 3:** Water variables in the three studied lakes.

Site		Depth	T °C	pH	TU	DO	TDS ug.L^–1^	N ug.L^–1^	P ug.L^–1^	Cond μS.cm^–1^	Clor-*a*
**FP**	Min	320	28.2	5.8	12.1	27.7	29	1612.8	20.3	0.04	-0.3
	Max	650	29.1	6.4	27.1	41.4	31	2822.4	36.8	0.05	2
	Mean	454	28.5	6.1	16.4	34.7	29.5	2096.6	26.9	0.05	0.77
	SD	111	0.3	0.2	4.2	5.3	0.7	395.1	5.4	0	1
	CV (%)	25	0.9	3.4	25.3	15.4	2.3	18.8	20	2.17	176
**BP**	Min	160	28.1	5.8	0	29.7	24	1478.4	16.6	0.04	0.68
	Max	380	29.5	6.7	47.2	55.4	32	2419.2	49.3	0.05	6.14
	Mean	216	28.5	6.2	21.8	41.2	28.8	1827.8	31.5	0.04	0.78
	SD	59	0.4	0.3	15.3	7.5	2.9	276.7	8.3	0	1.82
	CV (%)	27	1.4	4.4	70.3	18.2	10.2	15.1	26.4	9.86	231.74
**CP**	Min	120	29.2	5.9	5.5	48.6	23	1478.4	13	0.04	0.34
	Max	270	30.2	6.8	61.6	93.3	38	2688	41.9	0.06	3.41
	Mean	194	29.6	6.4	38.6	62.7	28.1	1948.8	23.9	0.04	0.51
	SD	48	0.3	0.3	13.4	13	4.8	442.7	10.2	0.01	1.05
	CV (%)	25	0.9	4.7	34.7	20.7	17	22.7	42.7	17.14	204.94

^*a*^Ferradura lake (FP), Burro lake (BP) and Caracará lake (CP). Minimum value (Min), maximum value (Max), mean value (Mean), standard deviation (SD), coefficient of variation (CV%).

^b^Depth collect (cm); water temperature (T °C); turbidity (TU); dissolved oxygen (DO %); total dissolved solids (TDS ug.L^–1^); total nitrogen (N μg.L^–1^); total phosphorus (P μg.L^–1^); electrical conductivity (Cond μS.cm^–1^); chlorophyll-*a* (Clor-*a* ug.L^–1^).

The three lakes showed high total N concentrations, according to CONAMA resolution for the protection of aquatic communities, with the maximum level in Ferradura lake. The high N concentrations in the water, compared to P, increased the N:P ratio at all points, with high mean values (Ferradura: 79:1, Burro: 63:1 and Caracará: 94:1), with the maximum value observed in Caracará (217:1), as evidenced by the low concentrations of total P (FP = 2.05; BP = 6.14; CP = 3.41 ug/L^-1^) and by the low chlorophyll-a concentration.

Pearson analysis showed that depth had a negative correlation with water temperature, pH, DO % and TSI, and the TU positive with the water temperature with the pH and DO % (Table 4).

**Table 4:** Correlation coefficients (Pearson) between abiotic variables.

Variables	Depth	T °C	pH	TU	DO%	TN μg.L^–1^	TP μg.L^–1^	TSI
Depth cm	**1**	**-0.46**	**-0.43**	-0.32	**-0.54**	0.2	0	**-0.71**
T °C	**-0.46**	**1**	**0.43**	**0.54**	**0.79**	-0.03	-0.3	0.08
pH	**-0.43**	**0.43**	**1**	**0.65**	**0.41**	-0.02	-0.24	0.27
TU	-0.32	**0.54**	**0.65**	**1**	**0.42**	-0.03	-0.1	0.24
DO%	**-0.54**	**0.79**	**0.41**	**0.42**	**1**	-0.2	**-0.38**	0.21
N μg.L^–1^	0.2	-0.03	-0.02	-0.03	-0.2	**1**	0.23	-0.2
P μg.L^–1^	0	-0.3	-0.24	-0.1	**-0.38**	0.23	**1**	**0.4**
TSI	**-0.71**	0.08	0.27	0.24	0.21	-0.2	**0.4**	**1**

^a^Depth collect (cm); water temperature (T °C); turbidity (TU); dissolved oxygen (DO %); total nitrogen (TN μg.L^–1^); total phosphorus (TP μg.L^–1^); trophic status index (TSI).

^b^Correlations in bold are significant at p <0.05 N = 30.

PCA analysis using only the significant abiotic variables (Pearson), explained a total of 61.95% of the total variation in the two first axes.

**Fig 1:**
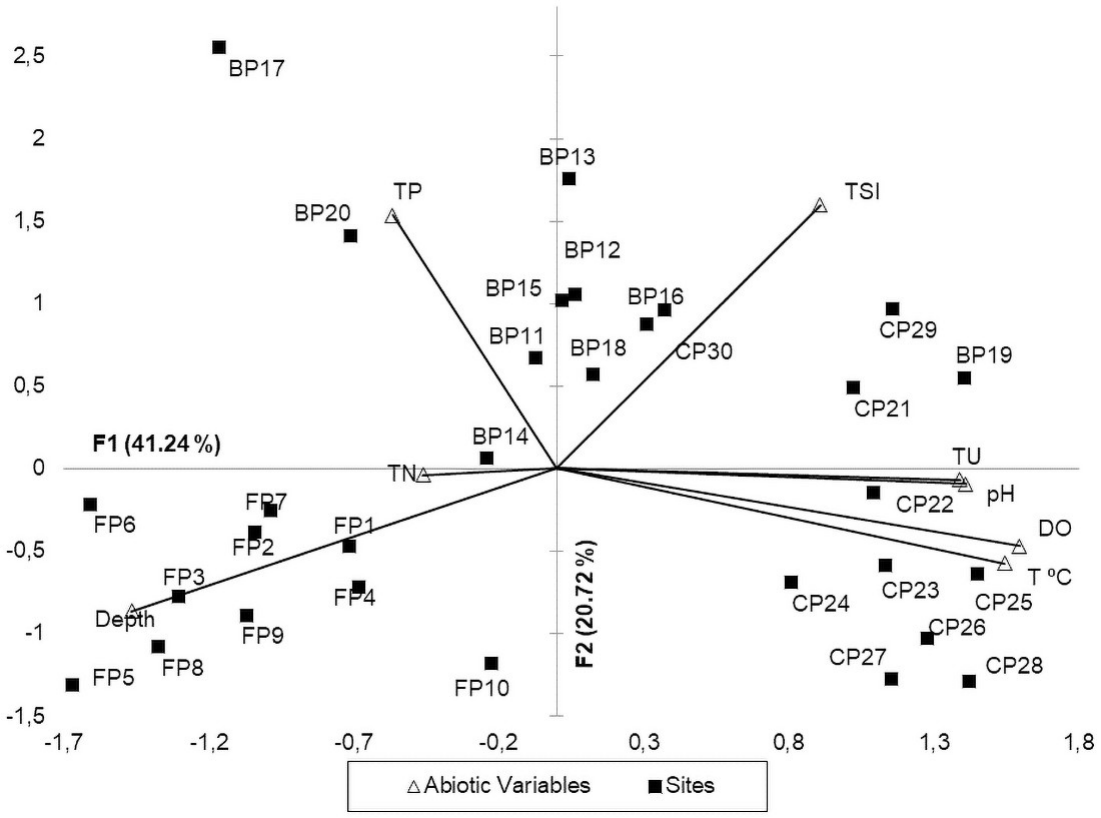
Principal Component Analysis (PCA) with 8 abiotic variables that presented correlation and 30 sites of the lakes. Depth collects; water temperature (T °C); hydrogenation potential (pH); Dissolved oxygen (DO%); Total Nitrogen for water (TN); Total phosphorus for water (TP) and Trophic State Index (TSI), Ferradura (FP) Burro (BP) and Caracará (CP).

The first axis (F1) recorded an explicability of 41.24%, with emphasis on total N and depth, with higher contributions of their negative scores, and greater positive contributions to the level of DO %, T °C, pH and TU. These contributions distinguished the Caracará and Ferradura lakes. In the Caracará lake, the higher water temperature were observed together with the higher values of the variables TU, DO % and pH, and concomitantly with the lower depth. In Ferradura lake the higher the depth and total N, the lower the values of TU, DO %, pH, and water temperature (Fig 1).

The second axis (F2) recorded an explicability of 20.72%, with higher contributions of total P and higher trophic state, separating BP from the other two lakes. The Burro lake is shallow in depth, with the highest level of total P and consequently higher trophic level (Fig 1).

### Index of Trophic Status

**Fig 2:**
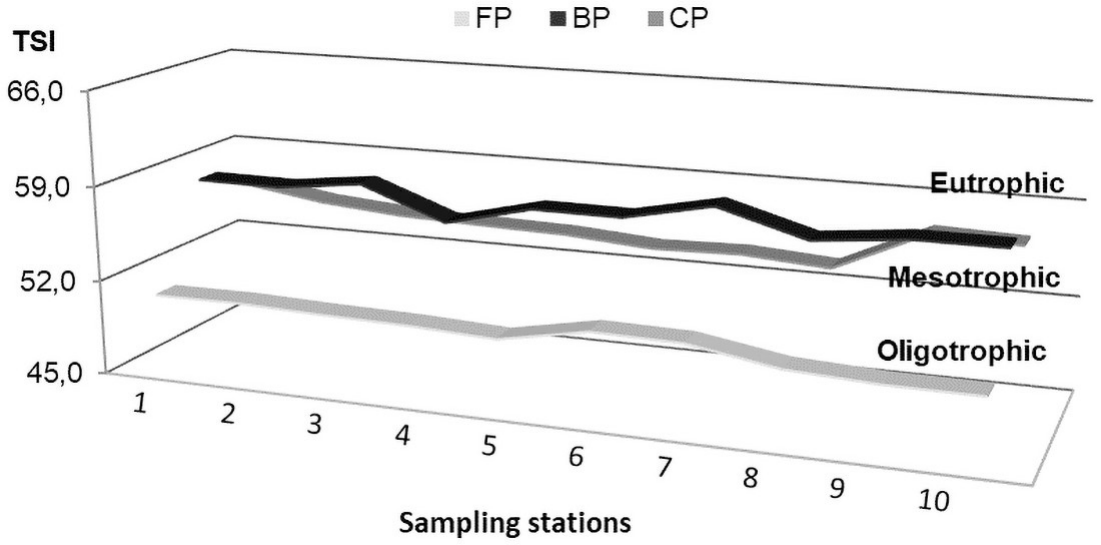
Trophic level, classification of the *Cetesb* in relation to the Trophic Status Index (TSI). Ferradura lake (FP) oligotrophic (47≤ TSI ≤ 52) minimum trophic level, Burro lake (BP) and Caracará lake (CP), both mesotrophic (52 ≤ TSI ≤ 59), medium trophic level.

Index of Trophic Status was evaluated by the determination of chlorophyll-a, considered to be the response of the water body to the causative agent [69]. Therefore, the Ferradura lake presented an oligotrophic state and both the lakes, Burro and Caracará, presented a mesotrophic state, according to the Cetesb-TSI classification [69]. The three lakes did not present enrichment due to excess nutrients and they are between low and medium values of the acceptable parameters of the trophic state index (Fig 2).

### Biotic variables

In the surface sediments of the three lakes, we found 119 taxa belonging to 31 genera, with greater richness of the genus *Eunotia* Ehrenberg (40 taxa) and greater abundance of *Aulacoseira* Thwaites. Moreover, 26 taxa were common to the three lakes. The Ferradura lake presented the greatest richness, with a total of 82 taxa, Caracará lake with 77 taxa and the Burro lake with 71 taxa. None of the taxa was dominant (+50%) and 25 showed abundance greater than 5% according to the relative density analysis (Table 5).

**Table 5:** Codes and denomination of species with more than 5% abundance in the samples collected from the three lakes under study.

Code	Denomination of the Species	FP	BP	CP
AEXG	*Achnanthes exiguum* (Grun) Czarnecki	x	x	
AAMB	*Aulacoseira ambigua* (Grun.) Simonsen	x	x	x
AUGR	*A*. *granulata* (Ehr.) Simonsen	x	x	x
AUIT	*A*. *italica* (Ehr.) Simonsen	x	x	x
AUPU	*A*. *pusilla* (Meister) Tuji & Houki		x	
ASIM	*A*. *simoniae* Tremarin, Torgan & T.Ludwig		x	
AUMI	*A*. *minuscula* Tremarin, Torgan & T.Ludwig		x	
AUVE	*A*. *veraluciae* Tremarin, Torgan & T.Ludwig	x	x	x
ECUT	*Eunotia curtiraphe* Metzeltin & Lange-Bertalot	x	x	x
EDID	*E*. *didyma* Grunow	x	x	x
EDMG	*E*. *desmogonioides* Metzeltin & Lange-Bertalot	x		x
EFOR	*E*. *formica* Ehrenberg	x	x	x
ELGC	*E*. *longicamelus* Costa, Bicudo & Wetzel	x	x	x
EMET	*E*. *metamonodon* Lange-Bertalot	x	x	x
EMON	*E*. *monodon* Ehrenberg	x	x	x
EFLX	*E*. *flexuosa* (Brébisson) Kützing	x		
EPAP	*E*. *papilio* (Ehr.) Hustedt	x	x	
EGUI	*E*. *guianense* (Ehr.) De Toni	x	x	x
EPAN	*E*. *pantropica* Glushchenko, Kulikovskiy & Kociolek	x	x	x
ETRA	*E*. *transfuga* Metzeltin & Lange-Bertalot	x	x	x
FBRA	*Fragilariforma brasiliensis* (Grun.) P.D.Almeida, C.E.Wetzel, E.Morales & D.C.Bicudo	x	x	x
PSYM	*Placoneis symmetrica* (Hust.) Lange-Bertalot	x	x	x
SLCR	*Staurosirella crassa* (Metz. & Lange-Bertalot) Ribeiro & Torgan	x	x	
SLDB	*S*. *dubia* (Grun.) Morales & Manoylov		x	
SGOU	*Synedra goulardii* Brébisson ex Cleve & Grunow	x		x

^a^Presence (x) and absence (-) of species in the lakes: Ferradura Lake (FP), Burro Lake (BP) and Caracará Lake (CP).

**Fig 3:**
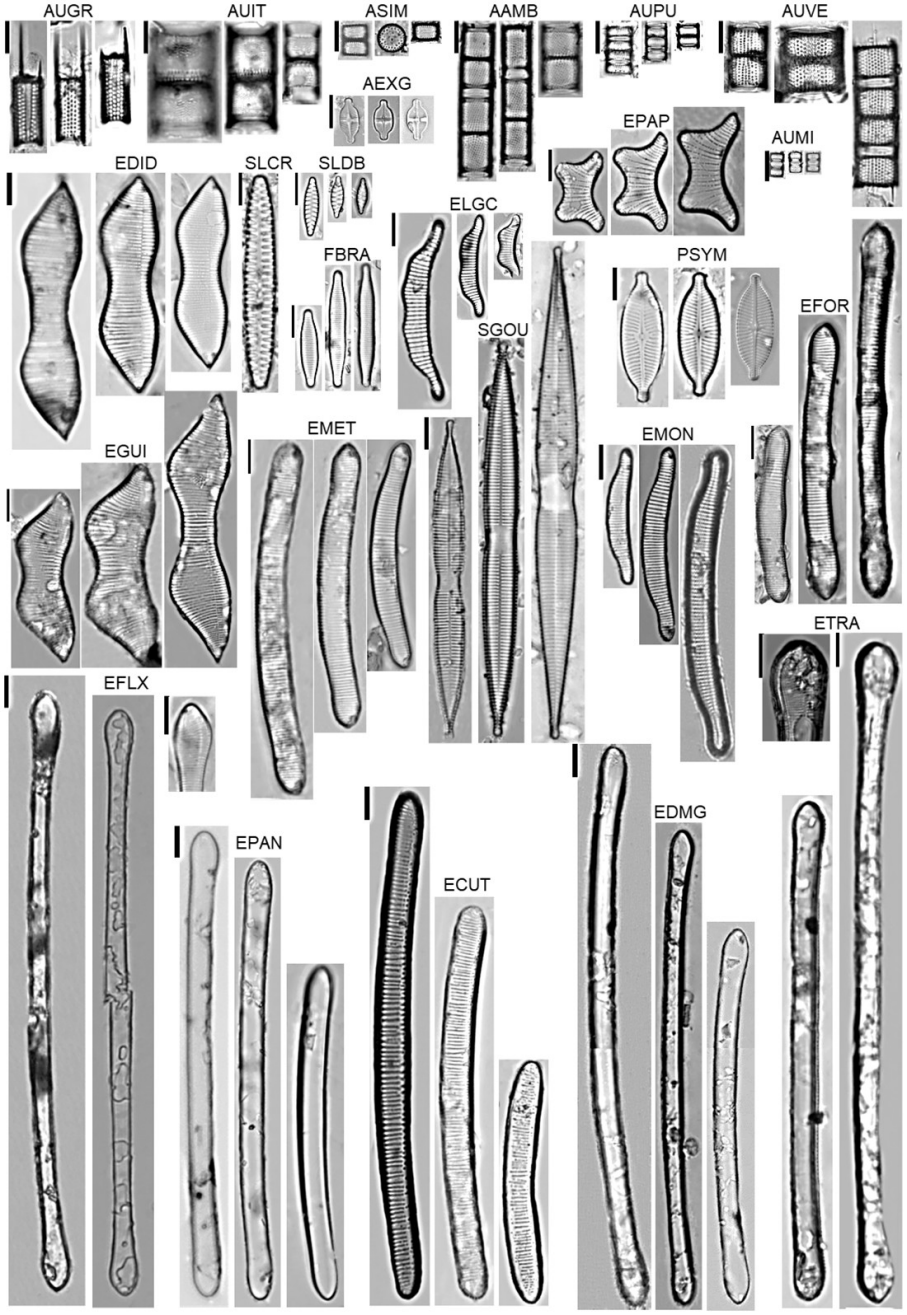
Illustration of the abundant species (> 5%) for the three lakes. AEXG: *Achnanthes exiguum*; AAMB: *Aulacoseira ambigua*; AUGR: *A*. *granulata*; AUIT: *A*. *italica*; AUPU: *A*. *pusilla*; ASIM: *A*. *simoniae*; AUMI: *A*.*minuscula*; AUVE: *A*. *veraluciae*; ECUT: *E*. *curtiraphe*; EDID: *E*. *didyma*; EDMG: *E*. *desmogonioides*; EFOR: *E*. *formica*; ELGC: *Eunotia longicamelus*; EMET: *E*. *metamonodon*; EMON: *E*. *monodon*; EFLX: *E*. *flexuosa*; EPAP: *E*. *papilio*; EGUI: *E*. *guianense*; EPAN: *E*. *pantropica*; ETRA: *E*. *transfuga*; FBRA: *Fragilariforma brasiliensis*; PSYM: *Placoneis symmetrica*; SLCR: *Staurosirella crassa*; SLDB: *S*. *dubia*; SGOU: *Synedra goulardii*.

The values of the descriptive analysis of the 25 species of the lakes, FP, BP and CP are presented in Table 6, illustration in Fig 3. The greatest abundance in the Ferradura lake was for *Aulacoseira italica* (AITA). In the Burro lake, the species *Eunotia transfuga* (ETRA), *Aulacoseira pusilla* (AUPU) and *A*. *veraluciae* (AUVE), and in the Caracará lake, *Eunotia desmogonioides* (EDMG).

**Table 6:** Correlation coefficients between abiotic and biotic variables.

Sites	FP				BP				CP			
^a^Species	^b^Max	^b^Mean	^b^SD	^b^CV (%)	^b^Max	^b^Mean	^b^SD	^b^CV (%)	^b^Max	^b^Mean	^b^SD	^b^CV (%)
AEXG	0.5	0	0.1	300	15	1.8	4.4	247	0	0	0	0
AAMB	0.2	0	0.1	300	12.4	3.5	3.8	110	3.3	0.4	1	277.1
AUGR	4	0.9	1.2	128.8	11.2	4.3	3	70.4	3.3	1.2	1.1	88.1
AUIT	49.6	39.8	7.8	19.7	15	4.8	4.5	93.4	11.8	4.5	3.1	69.7
AUMI	0	0	0	0	7.8	1.4	2.4	174.2	0	0	0	0
AUPU	0	0	0	0	44	6.1	12.9	211.5	0	0	0	0
ASIM	0	0	0	0	7.3	1.5	1.9	132	0	0	0	0
AUVE	5.5	0.9	1.8	208.6	40.2	19.1	13.3	69.7	2.3	1.2	0.5	38.2
ELGC	7	3.4	2.3	68	6	2.6	1.9	74.3	10	3.7	2.8	75.6
ECUT	6.5	1	1.9	185.3	4	0.8	1.3	165.8	22.5	5.4	6.6	122.1
EDID	5	1.4	1.6	107.5	2.5	0.5	0.9	201.5	2.5	0.9	0.8	89.8
EDMG	3.5	0.5	1.1	235.4	0	0	0	0	36	19.7	10.5	53.3
EFOR	23.4	11.7	7.8	66.6	33	11.6	10	86.4	11.5	6.9	3.3	47.6
EMET	15	7.7	4.6	59.8	5	1.5	1.7	114.5	8	2.5	2.7	107.3
EMON	13.3	5.3	3.9	73.2	4	1.1	1.2	104.6	14.5	4.7	3.8	80.7
EFLX	5	1	1.7	178.7	0	0	0	0	0	0	0	0
EPAP	2	0.4	0.8	200	6	1.6	2.2	140.9	0	0	0	0
EGUI	0.5	0.2	0.2	152.8	9	0.9	2.7	300	1.8	1	0.7	69.2
EPAN	18.5	7.1	5.1	71	22.6	7.2	7.2	99.6	30.7	20.6	6.4	31.2
ETRA	2	0.2	0.6	300	44.3	9	13.2	146	6	1.5	2	139.1
FBRA	1	0.1	0.3	300	12.4	1.5	3.6	236.2	7	1.4	2	148.8
PSYM	1	0.3	0.4	137.8	6	0.8	1.8	240.8	1.5	0.5	0.7	152.8
SLCR	2	0.5	0.7	160.6	17	2.2	5	227.1	0	0	0	0
SLDB	0	0	0	0	17.5	2.7	5.4	196.2	0	0	0	0
SGOU	0.3	0	0.1	300	0	0	0	0	7.5	2	2	100.3

^a^Biotic variables: Species codes are described in Table 5.

^b^Values of maximum, mean density of species (cells.mL^-1^), standard deviation (SD) and coefficient of variation (CV %), of the 25 most abundant species (> 5%) for the three lakes under study: Ferradura Lake (FP), Burro Lake (BP) and Caracará Lake (CP).

Of the most abundant species (> 5%), 21 presented significant correlation p <0.05, by Pearson correlation matrix (Table 7). *Aulacoseira italica*, *A*. *granulata* and *A*. *veraluciae* were the species with highest correlation with biotic variables. Among the species with significant correlation, 35% species had strong interactions, 46% species weak interactions and 15% moderate interactions, regarding the classification by Hinkle et al. [69].

**Table 7:** Correlation coefficients (Pearson) between biotic variables.

^b^**Species**	AEXG	AAMB	AUGR	AUIT	AUMI	AUPU	ASIM	AUVE	ECUT	EDMG	EMET	EMON	EFLX	EPAP	EPAN	ETRA	FBRA	PSYM	SLCR	SLDB	SGOU
AEXG	**1**	0.11	0.05	-0.17	0	-0.05	0.07	0.09	-0.12	-0.14	-0.13	-0.13	-0.06	-0.01	-0.26	-0.05	0.05	0.2	**0.98**	**0.94**	-0.1
AAMB	0.11	**1**	**0.69**	-0.36	0.13	0.11	0.22	**0.8**	-0.2	-0.25	-0.24	-0.3	-0.14	0.13	-0.18	0.19	**0.6**	-0.1	0.07	0.07	-0.17
AUGR	0.05	**0.69**	**1**	**-0.41**	0.35	0.34	**0.49**	**0.83**	-0.22	-0.35	-0.3	-0.23	-0.23	0.23	-0.31	**0.42**	0.2	0.23	0	0.14	-0.13
AUIT	-0.17	-0.36	**-0.41**	**1**	-0.17	-0.15	-0.24	**-0.41**	-0.19	**-0.4**	**0.64**	0.23	**0.5**	-0.1	-0.33	-0.28	-0.3	-0.08	-0.1	-0.21	-0.32
AUMI	0	0.13	0.35	-0.17	**1**	**0.95**	**0.9**	0.24	-0.11	-0.18	-0.09	-0.26	-0.09	0.22	-0.29	0.03	-0.07	-0.06	-0.02	0	-0.14
AUPU	-0.05	0.11	0.34	-0.15	**0.95**	**1**	**0.96**	0.16	-0.11	-0.16	-0.09	-0.23	-0.07	0.03	-0.27	0.03	-0.06	-0.08	-0.07	-0.05	-0.12
ASIM	0.07	0.22	**0.49**	-0.24	**0.9**	**0.96**	**1**	0.31	-0.16	-0.23	-0.18	-0.31	-0.11	0.09	-0.31	0.17	-0.06	0	0.06	0.1	-0.17
AUVE	0.09	**0.8**	**0.83**	**-0.41**	0.24	0.16	0.31	**1**	-0.22	-0.32	-0.34	**-0.4**	-0.18	**0.5**	-0.25	0.24	0.33	0.24	0.04	0.21	-0.23
ECUT	-0.12	-0.2	-0.22	-0.19	-0.11	-0.11	-0.16	-0.22	**1**	**0.52**	-0.11	0.06	-0.15	-0.14	0.35	-0.12	-0.16	-0.18	-0.13	-0.14	0.08
EDMG	-0.14	-0.25	-0.35	**-0.4**	-0.18	-0.16	-0.23	-0.32	**0.52**	**1**	-0.23	-0.02	-0.18	-0.26	**0.77**	-0.21	-0.06	-0.18	-0.18	-0.17	0.35
EMET	-0.13	-0.24	-0.3	**0.64**	-0.09	-0.09	-0.18	-0.34	-0.11	-0.23	**1**	0.3	**0.38**	-0.09	-0.29	-0.31	-0.24	-0.08	-0.04	-0.18	-0.18
EMON	-0.13	-0.3	-0.23	0.23	-0.26	-0.23	-0.31	**-0.4**	0.06	-0.02	0.3	**1**	**0.39**	-0.27	-0.05	-0.11	0.21	-0.13	-0.17	-0.19	**0.38**
EFLX	-0.06	-0.14	-0.23	**0.5**	-0.09	-0.07	-0.11	-0.18	-0.15	-0.18	**0.38**	**0.39**	**1**	-0.11	-0.03	-0.1	-0.07	-0.12	-0.08	-0.08	-0.13
EPAP	-0.01	0.13	0.23	-0.1	0.22	0.03	0.09	**0.5**	-0.14	-0.26	-0.09	-0.27	-0.11	**1**	-0.06	0.01	-0.08	0.16	-0.01	0.08	-0.2
EPAN	-0.26	-0.18	-0.31	-0.33	-0.29	-0.27	-0.31	-0.25	0.35	**0.77**	-0.29	-0.05	-0.03	-0.06	**1**	0.03	-0.13	-0.31	-0.29	-0.32	**0.4**
ETRA	-0.05	0.19	**0.42**	-0.28	0.03	0.03	0.17	0.24	-0.12	-0.21	-0.31	-0.11	-0.1	0.01	0.03	**1**	-0.06	0.1	-0.07	0.01	-0.1
FBRA	0.05	**0.6**	0.2	-0.3	-0.07	-0.06	-0.06	0.33	-0.16	-0.06	-0.24	0.21	-0.07	-0.08	-0.13	-0.06	**1**	0.02	0.01	0.02	0.19
PSYM	0.2	-0.1	0.23	-0.08	-0.06	-0.08	0	0.24	-0.18	-0.18	-0.08	-0.13	-0.12	0.16	-0.31	0.1	0.02	**1**	0.2	**0.49**	-0.04
SLCR	**0.98**	0.07	0	-0.1	-0.02	-0.07	0.06	0.04	-0.13	-0.18	-0.04	-0.17	-0.08	-0.01	-0.29	-0.07	0.01	0.2	**1**	**0.92**	-0.13
SLDB	**0.94**	0.07	0.14	-0.21	0	-0.05	0.1	0.21	-0.14	-0.17	-0.18	-0.19	-0.08	0.08	-0.32	0.01	0.02	**0.49**	**0.92**	**1**	-0.12
SGOU	-0.1	-0.17	-0.13	-0.32	-0.14	-0.12	-0.17	-0.23	0.08	0.35	-0.18	**0.38**	-0.13	-0.2	**0.4**	-0.1	0.19	-0.04	-0.13	-0.12	**1**

^a^Correlations in bold are significant at p <0.05.

^b^Biotic variables: Species codes are described in Table 5.

PCA using only the significant biotic variables (Pearson) showed an explicability of 38.58% considering the first and second axes: The first axis (F1) registered an explicability of 23.45%, with emphasis for six mero-planktonic species of the *Aulacoseira* genus (AUVE, AUGR, AAMB, ASIM, AUMI, AUPU) and three periphytic species (EPAP, ETRA, FBRA) with higher contributions of their positive scores, and greater negative contributions for four periphytic species, three of the *Eunotia* genus (ECUT, EDMG, EPAN) and one *Synedra* (SGOU).

The second axis (F2) recorded an explicability of 15.13%, with emphasis for three periphytic species (EFLX, EMET, EMON) and one mero-planktonic species (AUIT), with higher contributions of their negative scores, and greater positive contributions to four periphytic species (AEXG, SLCR, SLDB, PSYM).

**Fig 4:**
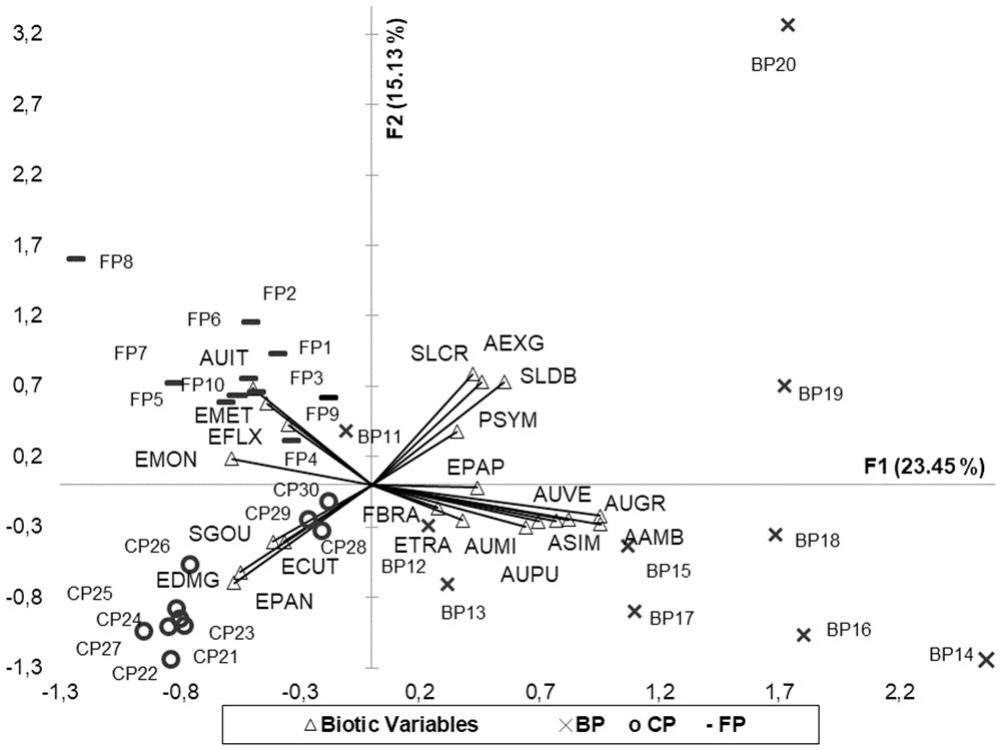
Principal Component Analysis (PCA) with 21 biotic variables, and 30 sites of the lakes: Ferradura (FP) Burro (BP) and Caracará (CP).

The positive scores of the first axis are driven primarily by planktonic species of the *Aulacoseira* genus, and the second axis is driven by periphytic species. The Burro lake presented greater richness of *Aulacoseira* species and Caracará and Ferradura lakes a richness of *Eunotia* species. The diatom sample scores of Principal Component Analysis (PCA) are shown in Fig 4.

### Biotic and abiotic correlations

Chemical and physical variables analyzed in the lakes had no significant differences (p <0.05). However, some variables showed different patterns, which allowed distinguishing the three lakes. Pearson’s correlation between biotic and abiotic variables resulted in 14 species presenting statistical significance (p <0.05) to perform analysis by CCA (Table 8).

**Table 8:** Correlation coefficients (Pearson) between biotic and abiotic variables.

Variables	Depth cm	T °C	pH	D0%	TN μg.L^–1^	TP μg.L^–1^	TSI
AAMB	-0.26	-0.358	-0.027	-0.066	-0.163	0.159	**0.458**
AUGR	**-0.372**	-0.253	0.14	-0.006	-0.116	0.161	**0.48**
AUIT	**0.851**	**-0.398**	**-0.453**	**-0.556**	0.226	0.047	**-0.821**
AUVE	-0.308	-0.328	0.074	-0.068	-0.297	0.222	**0.57**
ELGC	0.049	0.021	-0.146	-0.021	0.325	**0.392**	-0.002
EDID	0.296	0.071	-0.045	0.025	**0.483**	-0.059	-0.328
EDMG	-0.309	**0.638**	**0.493**	**0.561**	-0.207	-0.359	0.063
EMET	**0.578**	-0.234	-0.313	-0.345	0.149	-0.168	**-0.633**
EMON	0.237	0.152	-0.189	0.229	0.003	-0.069	**-0.4**
EFLX	**0.429**	-0.207	-0.301	-0.155	-0.094	0.014	**-0.364**
EPAP	-0.071	-0.268	-0.286	-0.238	-0.254	**0.365**	0.312
EPAN	-0.257	**0.533**	0.241	**0.438**	-0.073	-0.068	0.124
ETRA	-0.282	-0.13	0.174	-0.031	0.05	**0.403**	**0.443**
SGOU	-0.344	**0.693**	-0.044	**0.774**	-0.119	-0.198	0.064

^a^Correlations in bold are significant at p <0.05.

^b^Biotic variables: Species codes are described in Table 5.

^c^Abiotic variables: Depth (cm); water temperature (T°C); hydrogenation potential (pH); dissolved oxygen (DO%); total nitrogen for water (TN); total phosphorus for water (TP); and level of trophic status index (TSI).

**Fig 5:**
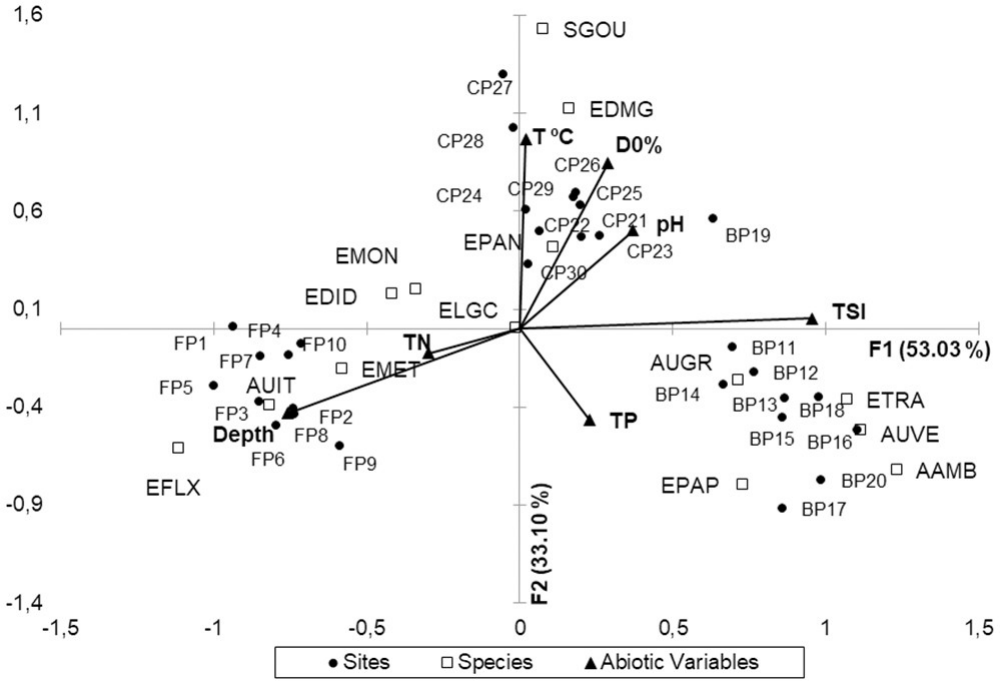
Ordination for CCA with 14 species (Table 8), 07 abiotic variables and 30 sites of the three lakes. Depth; water temperature (T °C); hydrogenation potential (pH); dissolved oxygen (DO%); total nitrogen (TN); total phosphorus (TP); trophic state index (TSI), Ferradura (FP) Burro (BP) and Caracará (CP).

CCA presented an explicability of 86.13% considering the first two ordination axes. The species matrix was linearly related to the abiotic variables (pseudo-F = 2.37) (*p* = 0.05) (Fig 5).

The first axis (F1) recorded an explicability of 53.03% (auto-value = 0.479), with higher contribution of N and depth for their negative scores, and higher positive contributions to the level of TSI and phosphorus.

In the Burro lake, three species of *Aulacoseira* Thwaites (AAMB, AUGR, AUVE) and two *Eunotia* Ehrenberg species (EPAP, ETRA) were present in high abundances when the level of trophic state and phosphorus were also high, and the depth was low. However, on the other side of the axis, for Ferradura lake, the higher the concentration of N in the water, the greater the depth, and with lower level of trophic state, the higher was the contribution of diatom species AUIT, EFLX, EMET, EDID and EMON. *Eunotia longicamelus* occurred in the three lakes, but in greater abundance in places with lower phosphorus level (Fig 5 and species codes in Table 5).

The second axis (F2) recorded an explicability of 33.10% (auto-value = 0.318). The variables that contributed most to the positive scores for this axis were the higher water temperature, higher availability of DO % and pH around 7.

The combinations of levels of these abiotic variables differentiated the Caracará lake from the other two lakes, with the highest abiotic values for pH, water temperature and DO % and a higher development of the three periphytic species (EPAN, EDMG, SGOU).

## Discussion

In terms of trophic conditions, the Ferradura lake is oligotrophic, while the lakes Burro and Caracará are mesotrophic, indicated by the highest concentration of phosphorus in both downstream lakes. Note that ours results, compared with data found in the literature to characterize the sites [12], seem not to support that the Cuiabá river is influenced by the discharge of sewage as well as by pisciculture that release their effluents.

However, according to studies by Yang et al. [70] available nutrients such as N and P seem to be the key point to control eutrophication and, under limiting conditions of one of the nutrients N or P, no significant increase in algae will occur in the water bodies. Some authors state that if the N:P ratio is greater than 10:1 [71] or 16:1 [72] P is acting as the limiting factor [73]. In the three lakes this was observed low total-P and also low chlorophyll-a concentration, but high concentration of N, in this case the P may be acting as a limiting factor. The higher level of N in the Ferradura lake, may be keeping the oligotrophy state for N:P ratio. The high N concentration in this lake comes from the discharge of effluent from fish farming tanks in the Cuiabá River [74].

The Burro lake presented the highest level of trophic conditions among the three lakes, but it is a lake with a lower depth, which receives greater discharge from the São Lourenço, a river impacted by gold mining activities [12]. This activity can compromise the quality and conservation of natural resources (water, soil, air) and affect the environment, primarily by using non-renewable resources and by altering the ecological balance. Analysed by Zeilhofer et al. [74] that studied the Northern Pantanal during the drought and flood phases and observed different phosphorus daily variation (1–4 t/day respectively) with higher P concentration in the São Lourenço river (0–5 t/day).

The three lakes did not present species dominance but, in general, *Aulacoseira* species had the greatest abundance and the *Eunotia* genus the greatest richness. Among the species with significant correlation, 35% of them had strong interactions and 46% had weak interactions regarding the classification by Hinkle et al. [75]. Poulin et al. [76] suggests that the difference in abundance between species is greater in communities characterized by weak interactions, while strong interactions may lead to greater evenness in the abundance of species. This was true for the three lakes that had a low uniformity and a higher percentage of weak interactions.

The Burro lake showed a greater richness of *Aulacoseira* species. These species can live part of their life cycle in benthic environments and with water turbulence, they re-suspend and increase their colonization capacity in the plankton [77,78]. Several studies have reported that *Aulacoseira* species excel in environments with a higher trophic level [33,79], occurring in the surface sediment with the highest abundance in eutrophic conditions [80] and increase their abundance in oligotrophic environments to mesotrophic [81].

Although the *Aulacoseira* has most of its species with a preference for a high concentration of total P, this characteristic may not be a general rule of the *Aulacoseira* genus. In this study *Aulacoseira italica* (Ehrenberg) Simonsen (AITA) presented a statistically significant relation for most of the abiotic variables, being negative for TSI and pH. *Aulacoseira italica* was found preferably in oligotrophic environments, cleaner or less turbid waters, or zones of low pollution. According to studies by Nakamoto et al. [82] *Aulacoseira italica* is more adapted to medium depth (maximum 12.0 m—mean 3.0 m), mild temperatures and scarce nutrients concentration. The species also has the characteristic to form spores and remains at rest in the sediment, like the other species of its genus [77,78] and returns to the surface if any event stimulates its growth [82]. Previous studies done in lake *Broa*, Brazil with similar environment characteristics to our study, the dominant species was *Melosira italica* (Ehrenberg) Kützing (= *Aulacoseira italica* (Ehr) Sim.) in the rainy summer period [82]. The identification consisted of spherical filaments typical of a resting cell (spore of resistance) as also verified in our study.

According to our studies the greater abundance of spores of *A*. *italica* was apparently due to rain events (Pantanal flooding) that increased nutrients dilution prior to sampling. With the conclusion of the rain period and in still waters, the spores may have increased in the sediment for the next event where the species resurfaces again. Pantanal is strongly related to the flood pulse, when autochthonous processes are a result of resuspension tactics in which sediments containing diatoms are made available in the pelagic environment [83]. Most of its records (*A*. *italica* spores) are still more common in fossil materials [84,85] and sites with some type of disturbance [86]. Siver and Kling [87] examined samples from 60 US and Canadian lakes for phytoplankton, surface sediment, and sediment from lower sections of gravity cores, and *A*. *italica* was more common in older sediment remains. Houk [88] and Genkal [89] report that *Aulacoseira italica* is a rare species in lakes and streams, but did not record the chemical parameters of the environment. However, in a study of plankton samples performed in the *Iguaçu* River, Brazil, *Aulacoseira italica* was an occasional taxon, occurring in only 1 out of 24 samples analysed during one year [90].

Although *Aulacoseira italica* occurred in the three lakes, the highest abundance (49.6) occurred in the Ferradura lake which presented the most acidic pH. The other two lakes presented much lower abundances (BP: 15.0 and CP: 11.8) and less acidic pH. Greater abundances of *Aulacoseira italica* was also observed in slightly acidic waters in other studies [83,91], and is reported to be a very rare species in alkaline waters [90]. The diatom flora associated with *A*. *italica* is also distinctive. The most commonly found genera include *Eunotia*, species essentially benthic and characteristic of a very different environment from the open water plankton of *A*. *ambigua* and *A*. *granulata* [77,92–94]. Ecology studies recorded in the "Catalogue of the main ecologic parameters of non-marine diatoms (in Portuguese)" [95], present a very extensive range of optimal developmental characteristics for the *Aulacoseira italica*, but some of these contradict current observations. This may be due to misidentification of *Aulacoseira italica* (AITA) with *Aulacoseira valida* (Grunow) Krammer (AVAL). When correctly identified *A*. *italica* is a valuable environmental indicator because it greatly differs from the habitat of other *Aulacoseira* taxa such as AUGR. The ecology of *A*. *italica* is not well known but clearly differs from the planktonic species [77].

Ferradura lake is oligotrophic and presented the greatest species richness, particularly of the *Eunotia* genus, due to higher acidity, depth and total N concentration. Studies carried out in the Amazon, Brazil, a variety of *Eunotia* species were found at pH values between 4.4 and 5.3, proving evidence that acid pH provides environmental conditions to develop a very particular diatom community dominated by specimens of Eunotiaceae [96]. Liu et al. [94] observed a great decrease of the number of *Eunotia* species between pH of 4.3 to 10.2 and temperature of 11°C to 22°C. The highest number of Eunotiaceae were found in a marsh with a pH of 4.3 to 6.5, with EPAP a rare species found only at pH 4.8. Compared to the low diversity of *Eunotia* (2 species) found by Santos et al. [45] for lakes in the Pantanal, with the majority of the lakes studied having alkaline waters, we can highlight that the highest occurrence of the genus in this study is actually due to more acidic environment.

It was observed that in wetland of the Pantanal of Mato Grosso, due to flooding and *Decoada* process, natural allochthonous and autochthonous material are deposited at the bottom of the lakes and this produces more acidic waters [12] that creates a friendly environment for *Eunotia* genus. The genus *Eunotia* prefers acidic environments so, both EPAP and ETRA are species usually found in wetlands, with an optimum pH development below seven. They live in still water, shallow lakes and high water temperatures (>30 °C) [95].

In sediment analysis by [97], it was reported that EMET and EMON species occurred together under oligotrophic conditions. The EFLX is also considered a rare species, but may present better development in swampy habitats, during summer, with pH-values ranging from 4,5 to 6,5 (acidophilus), being this its better characteristic [94,98].

The combinations of the higher water temperature, higher availability of % DO, pH around 7 and lower depth, separated the Caracará lake from the other two lakes and with higher development of three periphytic species (EPAN, EDMG, SGOU). Most species of the genus *Eunotia* are described as acidophilic with development in acidic water [99]. *Eunotia pantropica* Glushchenko, Kulikovskiy & Kociolek (EPAN = *E*. *rabenhorstiana* var *elongate* (R.M.Patrick) Metzeltin & Lange-Bertalot) is a species that develops at pH < 7, with its optimum below pH 5.5, as well as in water rich in humic substances and temperatures above 30 °C [92].

Although in the Caracará lake the pH variation was between 5.9–6.8, the highest development of this species (EPAN) occurred in this lake in a site with pH variation between 6.4–6.8. Probably the low depth, high light availability and high water temperature of Caracará lake favoured the higher abundance of the species (59%) compared to the other two lakes (BP: 20.63% and FP: 20.34%). Besides the higher water temperature, the Caracará lake presented higher concentration of humic substances, and higher DO concentration. *Eunotia desmogonioides* (EDMG) is also a species that equals the ecology of EPAN, described above. In sediment studies by Faustino et al. [97] the same was observed in 23% of the samples under oligotrophic conditions. In other studies, the same occurred in oligo-mesotrophic waters, slightly acidic to neutral, characterizing the bio-indication of this species [100].

The third species with the greatest development in Caracará lake is *Synedra goulardii* (SGOU), an alkaliphilic epileptic species with an optimum development at pH around 7 [101]. In studies conducted by Nardelli et al. [102] this species also occurred at pH around 7 and in an environment of greater transparency and high availability of DO. It could be characterized that this species has a better development in waters with high concentration of DO.

Diatoms seemed to be a good bioindicator to evaluate the quality of water in the lakes studied. Diatom communities have been recommended by researchers in many countries as appropriate to the evaluation of water quality [103–108]. Several studies present a broad discussion about the use of diatoms, considering sensitivity especially to pH, conductivity, nutrient concentration, organic matter and dissolved oxygen [32,35–37,109–113]. Complementing, surface sediment diatoms are robust indicators of environmental conditions, strongly associated with environmental factors, probably because they integrate information in space and time [114].

In summary the three lakes showed a high diatom diversity, with numerous populations of *Eunotia* and *Aulacoseira* genera. The wetlands often presented low pH which provides environmental conditions to develop the *Eunotia* genus. This was true in the Ferradura lake with more acidic environment. However, *Aulacoseira* genus, with three species (AAMB, AUGR, AUVE), characterised the environment towards trophic level increase. This was observed in the Burro and Caracará lakes that had a mesotrophic environment and with the mentioned species above as the most abundant. These species were a good indicator of trophic levels. On the other hand, *A*. *italica* was the most important species under oligotrophic environment in the lake Ferradura, with more acidic environment.

Despite the greater abundance of *Eunotia* and *Aulacoseira*, in different situations, there were no dominance species in the three lakes. This means that optimal conditions for the development of these species were not present, although favourable conditions promoted an increase in their populations. Lakes characteristics, slightly acidic water and oligotrophic to mesotrophic conditions, justified the diversity of diatom species group found in the sediments analysed (*Eunotia—*slightly acidic water and *Aulacoseira—*trophic condition), showing that diatoms are a good bioindicator, due to their sensitivity to physical and chemical variations in the environment. The species group have shown to be dependent on the concentration but also on the combination of physical and chemical parameters, that determine variations in the size of their populations.

Although the analysis of the average physical and chemical parameters in the water did not show significant differences among the three lakes, the combination of these parameters determined biotic differences in the lakes. Due to the richness found and the fact that we did not find teratological frustules, it can be inferred that the anthropogenic disturbances were still small in these three lakes. We hypothesize that the material accumulated downstream of the rivers also accumulated in the lakes and the diversity decreases due to mesotrophic conditions, however the differences between the lakes were still not extreme, as seen by biotic and abiotic conditions. Thus, the set of this information indicates that the studied environments of the Pantanal presented good ecological conditions from a naturally oligotrophic to mesotrophic environment. However, the results of this study in the context of the environmental destruction that the Pantanal underwent (fire/forest 2020) and the influence that it may have had on limnological processes, is of extremely relevance to raise new comparative studies for the area.
